# Ultra-extensible ribbon-like magnetic microswarm

**DOI:** 10.1038/s41467-018-05749-6

**Published:** 2018-08-21

**Authors:** Jiangfan Yu, Ben Wang, Xingzhou Du, Qianqian Wang, Li Zhang

**Affiliations:** 1 Department of Mechanical and Automation Engineering, The Chinese University of Hong Kong, Shatin, N.T., Hong Kong, 999077 China; 2 Department of Biomedical Engineering, The Chinese University of Hong Kong, Shatin, N.T., Hong Kong, 999077 China; 3 Chow Yuk Ho Technology Centre for Innovative Medicine, The Chinese University of Hong Kong, Shatin, N.T., Hong Kong, 999077 China; 4 T-Stone Robotics Institute, the Chinese University of Hong Kong, Shatin, N.T., Hong Kong, 999077 China

## Abstract

Various types of structures self-organised by animals exist in nature, such as bird flocks and insect swarms, which stem from the local communications of vast numbers of limited individuals. Through the designing of algorithms and wireless communication, robotic systems can emulate some complex swarm structures in nature. However, creating a swarming robotic system at the microscale that embodies functional collective behaviours remains a challenge. Herein, we report a strategy to reconfigure paramagnetic nanoparticles into ribbon-like swarms using oscillating magnetic fields, and the mechanisms are analysed. By tuning the input fields, the microswarm can perform a reversible elongation with an extremely high aspect ratio, as well as splitting and merging. Moreover, we investigate the behaviours of the microswarm when it encounters solid boundaries, and demonstrate that under navigation, the colloidal microswarm passes through confined channel networks towards multiple targets with high access rates and high swarming pattern stability.

## Abstract (web summary)

Manipulation of paramagnetic microparticles can be exploited for drug delivery. Here the authors manipulate a swarm of such particles and control its shape with a magnetic field so that it can elongate reversibly, split into smaller swarms and thus be guided through a maze with multiple parallel channels.

## Introduction

In nature, thousands or even millions of individual elements can form a wide range of patterns, purely through local communications, such as bacteria colonies^1,2^, bird flocks, and insect swarms^3^. Through collective pattern formation, these elements can dramatically change the swarming shape according to the environment they interact with. In the field of robotics, various types of robotic systems have been reported with swarm intelligence^4,5^, which are inspired to emulate part of the swarm behaviours in nature. More recently, a thousand-robot swarm capable of programmable self-assembly has been reported^6^, addressing both the physical and algorithmic challenges of a large-scale robotic swarm. These studies rely on wireless communications to plan and distribute each robot; however, at small scales, this method is hardly accessible due to the challenges of integrating onboard processors, sensors and actuators. Hence, different strategies are required for the design and development of artificial swarms at the micro/nanoscale. Colloids are promising candidates for understanding the guiding principles of swarm behaviours in living systems, and physical or chemical interactions among them may be considered as ‘communications’^7,8^. These materials play an important role as building blocks for creating complex systems via static and dynamic self-assembly processes, such as periodic crystals^9–13^, self-assembled colloidal devices^14–18^, clustering^19,20^ and flocking^21^, which may help us to understand the guiding principles of swarm behaviours in living systems. Nevertheless, emulating the swarm behaviours in nature is still challenging, because the relevant fundamental mechanisms, agent–agent interactions and proper actuation strategies are still under investigation. Moreover, realising collective morphological transformations that are similar to some living systems may require appropriate actuation methods and programmable interactions among the agents^22–24^.

In this paper, we trigger the formation of a microswarm on a 2-D plane, i.e., a reconfigurable ribbon-like paramagnetic nanoparticle swarm (RPNS) with a dynamic-equilibrium structure, by applying programmed oscillating magnetic fields. We investigate the generation mechanism and demonstrate the reversible elongation with an ultrahigh aspect ratio of the microswarm. Other reversible reconfigurations, including controlled splitting behaviours and the merging of two subswarms are presented. The microswarm can perform 2-D locomotion fully under control near a solid surface, and can maintain a stable pattern even in complex environments with varied boundary conditions. Finally, we demonstrate that the microswarm can pass through channel networks towards multiple targets with high access rates and perform non-contact micromanipulation in a fluid.

## Results

### Generation of a ribbon-like paramagnetic nanoparticle swarm

**Fig. 1:**
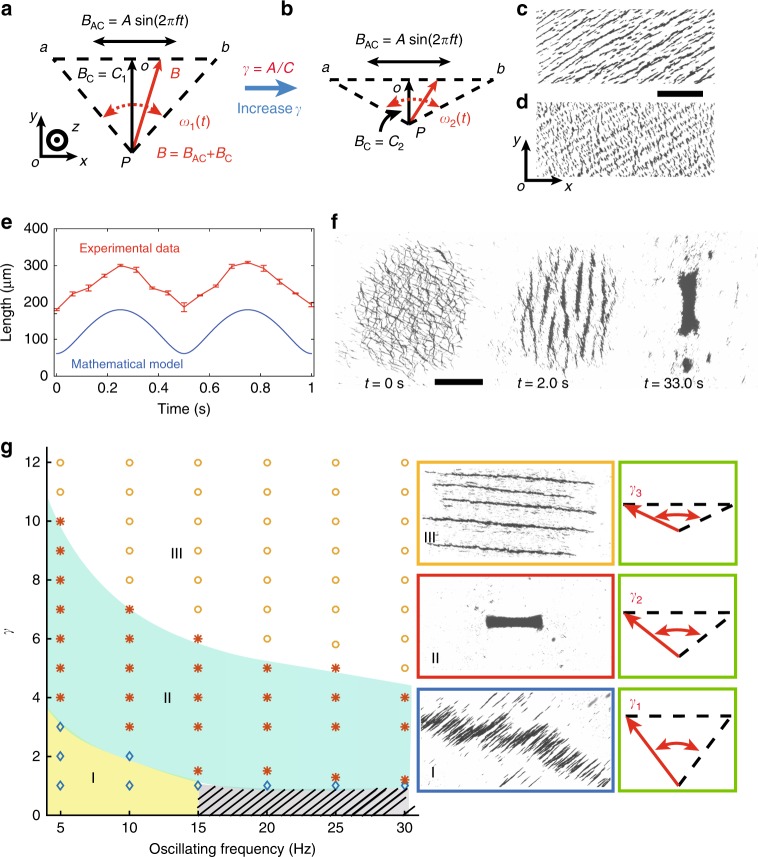
Actuation of ribbon-like magnetic microswarm using oscillating magnetic fields. **a** A schematic depiction illustrates the applied oscillating magnetic field. The red arrow shows the magnetic field, which is the superposition of *B*_C_ and *B*_AC_. The angular velocity of the field is represented by *ω*(*t*). The amplitude ratio is *γ* = *A*/*C*, and *A* is 10 mT in this paper. **b** A schematic depiction illustrates the oscillating magnetic field when the amplitude ratio is increased. **c** The particle chain formed when the field points *a* or *b*, in which case the field strength is the largest. The scale bar is 400 μm. **d** The particle chain formed when the field points *O*, in which case the field strength is the weakest. In **c** and **d** the applied oscillating frequency is 3 Hz. **e** The relationship between the length of the particle chains and time, actuated by the oscillating magnetic field. Each data point represents the average of 3 experiments. The error bar indicates the standard deviation (s.d.). **f** The generation process of an RPNS (Supplementary Movie 1). The scale bar is 800 μm. **g** The phase diagram showing the swarm patterns actuated by different input magnetic fields. The oscillating fields that trigger different swarm patterns are schematically presented in the green rectangles. In the grey shadowed region, no regular patterns can be formed

The oscillating magnetic field *B* for the actuation is schematically demonstrated (Fig. 1a). In one direction, an alternating magnetic field *B*_AC_ is applied, with the condition of *B*_AC_ = *A* sin(2*πft*), where *A* is the amplitude of the magnetic field as a constant, and *f* is the input oscillating frequency. The uniform magnetic field *B*_C_ is applied in the perpendicular direction with a constant field strength of *C*. An amplitude ratio (*γ* = *A*/*C*) is proposed. The superposed magnetic field (Fig. 1a, red arrow) has a time-dependent angular velocity and field strength. At Point *O*, the magnitude of the angular velocity is maximal, and the magnitude of the field strength is minimal (Supplementary Fig. 1a). When the amplitude ratio *γ* is increased, as shown in Fig. 1b, the oscillating angle becomes larger, and if the oscillating frequency is maintained, the angular velocity is also increased (*ω*_2_(*t*) > *ω*_1_(*t*)). Meanwhile, because the magnitude of *B*_AC_ is fixed, *C*_2_ becomes smaller than *C*_1_. In magnetic fields, paramagnetic nanoparticles form chain-like structures; therefore, we regard the individual nanoparticle chains as the building blocks in this work. Supplementary Fig. 2 illustrates the forces and torques exerted on a particle chain when it oscillates with the input field. The lengths of the particle chains are related to the strength of the applied field^25^. When the superposed field points to *a* or *b*, the magnetic field strength reaches the highest value, which enhances the magnetic attractive interactions between short nanoparticle chains, and longer chains are formed (Fig. 1c). The magnetic field strength is the weakest when the field points to *O*, and at this moment, the particle chains are much shorter (Fig. 1d). Figure 1e shows the change in the time-dependent chain lengths, when the oscillation frequency is 1 Hz. The blue and red curves indicate the mathematical model (the model is presented in Supplementary Eq. 3), and the experimental data, respectively, which demonstrates good agreement. Because in the model, a single particle chain is assumed to be formed, while in the experiments, chain-like bundles are formed due to the interactions between the adjacent chains. The particle–particle attraction forces in the experiments will be larger than that in the model, and the chains are more stable. Therefore, longer chains are formed in the experiments. Supplementary Fig. 1b demonstrates the magnetic interactions between the particle chains.

The generation process of an RPNS is demonstrated in Fig. 1f and Supplementary Movie 1. Initially, the nanoparticle chains are dispersed and distributed uniformly. When the oscillating magnetic field is applied, the nanoparticles locally gather into dynamic structures in a short time (*t* = 2.0 s). After a series of self-merging processes among subswarms are performed (Supplementary Fig. 3), a dynamically stable ribbon-like microswarm is generated (*t* = 33.0 s). In fact, the applied oscillating magnetic field can significantly modify the collective behaviours of the paramagnetic nanoparticles. Figure 1g presents the phase diagram, which indicates the relationship between the swarm patterns and input magnetic fields (i.e., the oscillating frequency and amplitude ratio *γ*). When *γ* is low, as shown in region I, the nanoparticles form uncontrollable massive chain-like patterns, which are unstable and tends to elongate. When *γ* increases (region II), a dynamic-equilibrium RPNS can be generated. Multiple clusters with long chain-like patterns are formed if the nanoparticles are actuated by the fields in region III. The insets in the green squares indicate the corresponding oscillating magnetic fields with a change in the amplitude ratio *γ*. No regular collective patterns can be formed in the grey shadowed region.

**Fig. 2:**
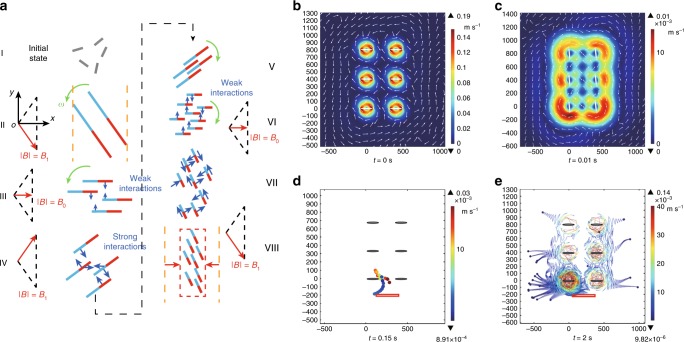
Schematic explanation of the swarm generation and simulation results of hydrodynamic signature. **a** The schematic generation process of an RPNS (Supplementary Movie 2). The rods illustrate nanoparticle chains, and the blue and red parts show the different magnetisations. **b**, **c** The simulation results of the flow field induced by an array of oscillating spheroids at *t* = 0 s and *t* = 0.01 s. The input amplitude ratio is 3, and the oscillating frequency is 20 Hz. **d** The simulation results of the motion of 30 free particles with a diameter of 10 μm at the tip of the array, at *t* = 0.15 s. The particles are released at *t* = 0 s, and are released from the red rectangle. **e** The trajectories of the particles at *t* = 2 s

The schematic explanation of the generation process is shown in Fig. 2a and Supplementary Movie 2. The rods indicate paramagnetic nanoparticle chains, and the red and blue parts represent the magnetisations of the chains. In stage I, dispersed nanoparticle chains are indicated by the grey rods, and the distribution is irregular because no magnetic field is applied at this moment. At the beginning of stage II, the oscillating field is applied. We take the initial situation with two long particle chains as an example, and the mechanism is applicable for cases when more chains are originally formed. The chains oscillate with the magnetic field (see the left part of stage II), and the oscillating direction at that moment is shown by the green arrows. The distribution of the chains along the *x*-axis is shown by the yellow dotted lines. The long chains are broken into several shorter ones during stage III and, due to the large angular velocity and small magnetic field strength in this stage, it is reasonable to assume that the magnetic interactions are not sufficiently strong to actuate the particle chains. Therefore, the locations of the centres of each split chains are maintained, until the field reaches the maximal oscillating amplitude (stage IV). Then, the chain-chain interactions become stronger with the increasing field strength, and the chains attract each other, making their distribution along *x*-axis narrower, as shown in stage IV and V of Fig. 2a. In these two stages, more of the surrounding particle chains will be attracted. Another half cycle is shown in stage VI–VIII. As a result, the chains are disassembled into much shorter ones, and through the reconfiguration induced by the chain-chain magnetic attractions, a more compact swarm pattern is formed in stage VIII. The ribbon-like pattern is shown by the dotted rectangle, and the shrinkage along *x*-axis is significant, which is labelled by the red arrows.

Fluidic interaction is another critical factor to trigger the formation of the swarm pattern. The fluidic velocity field induced by an oscillating chain has been analysed and simulated in Supplementary Figs. 4, 5. The flow profile induced by an RPNS is simulated, by regarding the swarm as an array of oscillating spheroids (Fig. 2b, c). The flow field reaches the maximal magnitude at 0 s, when the angular velocity of the spheroids is at the largest value. The strong flow induced will gradually dissipate with time, as shown at *t* = 0.01 s. As a result, the induced flow always surrounds the swarm pattern, avoiding the microswarm from contact with external objects and, therefore, enhances its pattern stability as an entity. Figure 2d, e shows the simulation results of the free-particle responses inside the flow field. The particles are firstly attracted into the microswarm and then are pumped out at the locations near the tip of the swarm, judging from the trajectories (Fig. 2e). The induced flow field has a repulsive effect to the agents along the long sides of the swarm. As a result, the dynamically stable pattern of the swarm is maintained, mainly due to the equilibrium of the repulsive fluidic interactions and the attractive magnetic interactions. Furthermore, the configurations of particle chains at the tips of the microswarm and its middle parts are investigated, which are schematically explained in Supplementary Figs. 6, 7.

During the generation process of an RPNS, the magnetic interaction among the particle chains is the main factor for the reconfiguration based on the analysis. Meanwhile, fluid drag is the reason for the disassembly of the particle chains when the magnetic field is reduced. Our strategy for the generation of an RPNS will remain effective if the fluidic drag torque is able to disassemble the chains, when the strength of the magnetic field reaches the minimum value. Therefore, the RPNSs can be scaled down until other particle–particle interactions (e.g., electrostatic forces, van der Waals forces and capillary forces) begin to play important roles to enhance the linking strength among particles. In this case, the drag torques are not sufficiently strong compared to the other interactions, and the particle chains remain almost the same length, which may hinder the formation of an RPNS. When the diameter of the nanoparticles is larger than 100 nm, our method remains effective. Supplementary Fig. 8 demonstrates the generation processes of RPNSs using nanoparticles with diameters of 100 nm and 250 nm, respectively. In addition, the pattern generation behaviours with different initial nanoparticle areal concentrations are investigated in Supplementary Figs. 9, 10.

### Multimodal reconfiguration

**Fig. 3:**
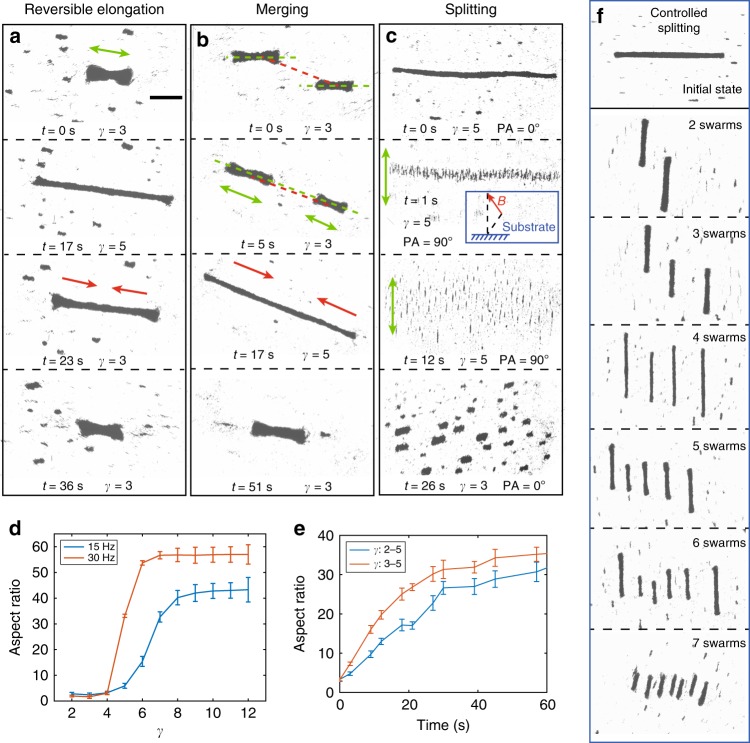
Pattern reconfiguration of the microswarm. **a** The demonstration of the reversible elongation of an RPNS (Supplementary Movie 3). The green arrow shows the direction of elongation, and the red arrows show the direction of contraction. **b** The merging process of the two self-governed RPNSs (Supplementary Movie 4). The long axis of the RPNSs are labelled by green dashed lines, and the linking between the centres of the swarms are indicated by the red dashed line. The directions of elongation and contraction are labelled by green and red arrows, respectively. **c** The splitting process of an RPNS (Supplementary Movie 5). PA represents the pitch angle of the applied magnetic field. **d** The change in the aspect ratio with the amplitude ratio *γ*. **e** The relationship between the aspect ratio of an RPNS and time. The applied oscillation frequency is 30 Hz. The blue curve indicates the results when the amplitude ratio increases from 2 to 5; while the red curve indicates the results when the amplitude ratio increases from 3 to 5. In **d** and **e** each data point represents the average of 3 experiments. The error bar indicates the standard deviation (s.d.). The scale bar is 600 μm. **f** Controlled splitting of RPNSs

Compared with microrobots having rigid monolithic structures^23,26^, RPNSs have more morphological flexibility and degrees of freedom. The experimental results of reversible elongation are demonstrated (Fig. 3a and Supplementary Movie 3). An RPNS is formed at *t* = 0 s, actuated by the oscillating field with a strength of 10 mT and an amplitude ratio *γ* of 3. At *t* = 3 s, *γ* is increased to 5, and the RPNS begins to elongate. The green arrows indicate the direction of elongation. The original aspect ratio of the RPNS is approximately 4, and at *t* = 17 s, the aspect ratio increases to 22. Then, *γ* is tuned to 3 again, and the RPNS gradually contracts to the original pattern (Fig. 3a, 36 s).

In some cases, more than one RPNSs may be generated, especially when the concentration of the nanoparticles is low. Some subswarms may not be attracted to each other and cannot perform self-merging, because the long-range fluidic interactions are weak. Figure 3b and Supplementary Movie 4 show the controlled merging process of two RPNSs. Initially, two remote RPNSs are generated by applying the oscillating magnetic field (10 mT, *γ* = 3, 30 Hz) on nanoparticles with a low areal density (7.5 μg mm^−2^). We judge that the distance between the two swarms is beyond the effective range of fluidic interactions, because after more than one minute, the relative distance remains almost constant. At *t* = 5 s, the direction of the chain axes (Fig. 3b, the green dashed lines) are tuned to coincide with the link between the centres of the microswarms (Fig. 3b, the red dashed line). Then, the amplitude ratio *γ* is increased to 5, and the swarm patterns are significantly elongated at *t* = 13 s. After the microswarms are long enough to contact each other, they merge to form one entity. The merged pattern is capable of shrinking again when *γ* is reduced to 3, and finally, we realise the merging of two independent subswarms into a stable RPNS. The splitting behaviour of an RPNS can also be realised by tuning the magnetic field (Fig. 3c and Supplementary Movie 5). An RPNS is formed and elongated initially, and then the oscillating plane of the field is changed to be vertical to the experimental plane (Fig. 3c, *t* = 1 s, inset). The pattern swells significantly due to the magnetic repulsive forces induced between the nanoparticle chains (Fig. 3c, green arrows). Then, the oscillating plane is tuned to be parallel to the experimental plane (coincide with the substrate), and multiple particle swarms are formed.

The relationship between the aspect ratio of an RPNS *α* and amplitude ratio *γ* is presented (Fig. 3d). For a single curve, the aspect ratio *α* is almost identical when *γ* is low, and then increases significantly when the amplitude ratio exceeds the triggering ratio (*γ* = 4). Then, *α* levels out and will not increase with *γ*. Meanwhile, the largest aspect ratio increases with the applied oscillating frequency. To trigger elongation of an RPNS, the chains are required to be sufficiently disassembled for reconfiguration. Therefore, a larger amplitude ratio and a higher oscillating frequency enhance the reconfiguration process. When the oscillating frequency is 30 Hz, the change in the aspect ratio *α* with time is shown (Fig. 3e). All the curves increase rapidly when *γ* is tuned to be 5 and gradually level out. To better understand the reversible elongation of the RPNS, we first investigate the induced magnetic forces between neighbouring particle chains with different relative positions (Supplementary Fig. 11a, b), and the mechanisms of the reversible elongation of an RPNS are schematically shown in Supplementary Fig. 11c, d and Supplementary Movie 6.

Moreover, the controlled splitting of RPNSs is realised, as shown in Figure 3f. The RPNS can be split into 2–7 subswarms with relatively identical sizes in a controlled fashion. In some cases, the subswarms formed by the two tip parts of the original RPNS are longer than those formed by the middle parts, which is attributed to the different configurations of particles between the tip and the middle part of the swarm. The controlled splitting is realised by combining a rotating magnetic field with the applied oscillating field. After an RPNS is generated, the entire swarm is actuated to perform in-plane rotation with a constant angular velocity, which reaches a counterbalance between the magnetic actuating torque and the hydrodynamic drag torque. By tuning the rotating frequency, the RPNS can be split into different numbers of subswarms under control. The phase diagram presenting the relationship among the rotating frequency of the entire swarm, the amplitude ratio *γ* and the number of split subswarms, is shown in Supplementary Fig. 12.

### Navigated locomotion and pattern stability in confined environments

**Fig. 4:**
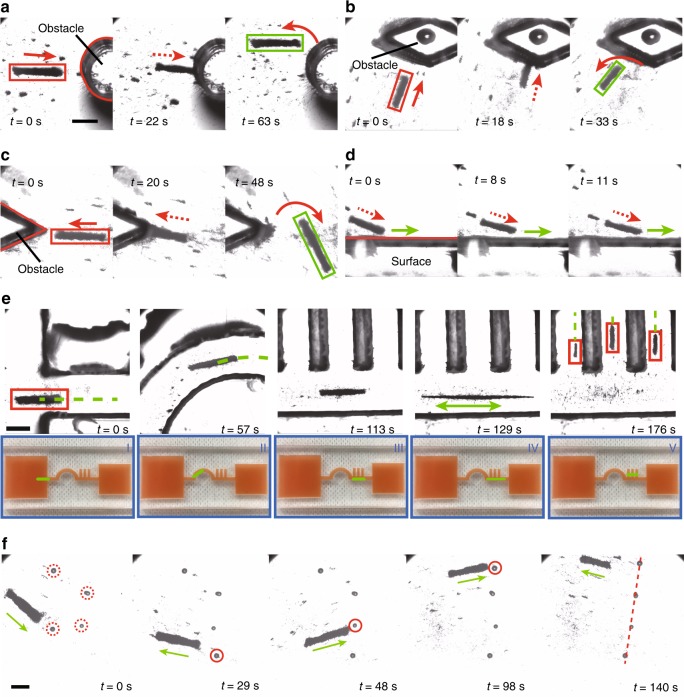
Stability, controlled locomotion and micromanipulation of the microswarm. **a**–**c** Circular and planar surfaces, and a sharp-angled pillar are applied as different boundaries, to investigate the behaviours of the RPNSs. **d** An RPNS moves along the direction tilted with a planar surface (Supplementary Movie 7). **e** The demonstration of an RPNS passing through a semi-circular channel, and accessing three targets (Supplementary Movie 8). The RPNS is highlighted by a red rectangle, and the green dotted lines indicate the direction of navigation. Images showing the overall view of the channel network are presented in the blue rectangles below each microscopic image, in which the green ribbons schematically represent the real-time position of the RPNS. **f** The non-contact manipulation of polystyrene microbeads using an RPNS (Supplementary Movie 9). The red dotted circles indicate the randomly distributed particles, and the green arrows represent the moving direction of the RPNS. The red circles show the particle that is being manipulated. In the demonstrations, the applied amplitude ratio during locomotion is 3.5 and that used for the pattern elongation is 6. The actuating pitch angle is 8°, and the oscillating frequency is 30 Hz. The scale bar is 800 μm

When an object approaches to a solid boundary, the drag coefficient will increase^27^. Using the mechanism, if a pitch angle is introduced into the actuating magnetic fields, the fluidic drag encountered by the upper and lower parts of the nanoparticle chains will be different. The translational motion of the entire microswarm is characterised in Supplementary Figs. 13, 14, and the mechanisms are explained in Supplementary Fig. 14c. In order to study the navigated motion of a microswarm in confined environments, we first investigate the behaviours of the microswarms with different boundary conditions, as shown in Fig. 4a–d and Supplementary Movie 7. An RPNS (in red rectangle) is steered to a cylindrical pillar and a planar surface, as shown in Fig. 4a, b, respectively. When it approaches the surface, the external field still actuates the swarm towards the direction of the red dotted arrows. After a short time, the RPNSs are guided to leave the pillars. During the process, the patterns are stable, and only a small part of the particles is lost, judging from the patterns (see in the green rectangles). The results in Fig. 4a, b demonstrate that the RPNS maintains its stability when encountering obstacles from the tips. The microswarm is also stable when an angled obstacle invades into it, as shown in Fig. 4c. Moreover, an RPNS with its moving direction tilted with a solid surface is demonstrated in Fig. 4d. The magnetic field actuates the swarm along the direction of the red dotted arrows and, as a result, the RPNS moves along the surface (the green arrows). Meanwhile, the swarm does not contact the surface during the locomotion due to the repulsive fluidic interaction with the surface. Therefore, based on the investigation, the RPNSs have an excellent pattern stability even in a complex environment with different boundaries, or with a flow speed in a submillimeter range.

Moreover, we demonstrate that an RPNS passes through a semi-circular channel and targets multiple destinations (Fig. 4e and Supplementary Movie 8). The channel consists of two parts: a semi-circular part and three branches serving as destinations. The real-time positions of the RPNS are labelled by the green rods in the overall views (Fig. 4e, blue rectangles). From 0 s to 88 s, the RPNS successfully passes through the semi-circular part of the channel. During locomotion, most of the nanoparticles are restricted inside the swarm, and the entire microswarm pattern keeps dynamically stable. After the RPNS approaches the entrance of the branches at *t* = 113 s, the amplitude ratio is increased to 6. A significant elongation is observed that the aspect ratio of the RPNS increases approximately from 6 to 28. Then, the input direction angle for the oscillation is increased to 90°, and multiple subswarms are formed. The subswarms can be actuated under control to enter all the three channel branches without the risk of structural collapse, due to their high pattern stability. Another demonstration is presented, that an RPNS splits into two subswarms, passes through two channels simultaneously, and finally remerges into one swarm (Supplementary Fig. 15). To further demonstrate the high-performance motion controllability and dexterity of the RPNS, the non-contact micromanipulation of four polystyrene microbeads using an RPNS as a robotic end-effector is performed (Fig. 4f and Supplementary Movie 9). The microbeads are distributed randomly (red dotted circles) and are one-by-one pushed by the flow generated at the tips of the RPNS. As a result, four microbeads are pick-and-placed to form a linear array. Moreover, if the average diameter of the microbeads is lower than 40 μm, some of the microbeads will not be pushed by the RPNS but be attracted into it (Supplementary Fig. 16 and Supplementary Movie 10).

**Fig. 5:**
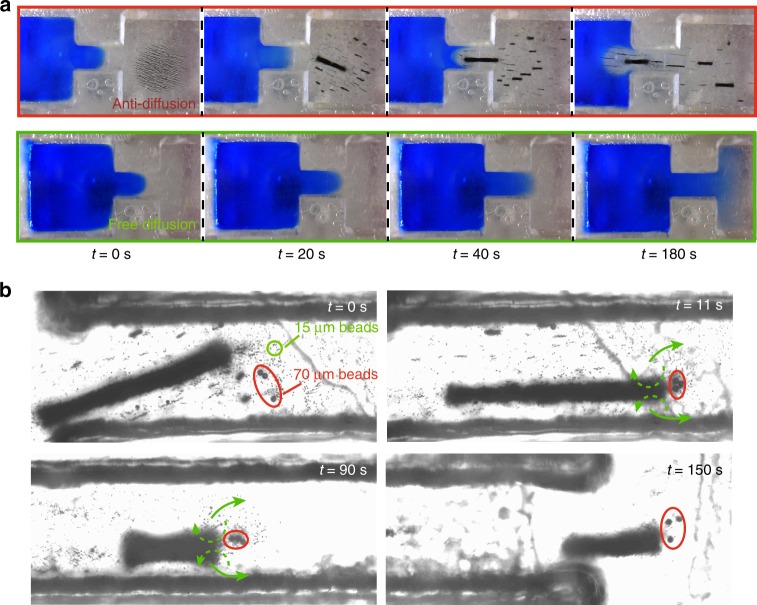
Anti-diffusion and sorting of non-magnetic beads using the microswarm. **a** The anti-diffusion effect induced by a ribbon-like microswarm in a microchannel. **b** Sorting 70 μm-diameter beads out from 15 μm-diameter beads in a channel using the microswarm. The 70 μm-diameter beads are labelled in the red ellipses, while some of the 15 μm-diameter beads are labelled in the green ellipse. One part of the small beads are pushed aside by the flow generated at the tip of the microswarm, as shown by the green arrows. The other part of the small beads are first attracted into the swarm, and then they are pumped out at some locations near the swarm tip, as shown by the green dotted arrows

Based on the fluidic flow induced by the tips of the microswarm, it may be applied as a tiny manipulation tool in microfluidic devices (Fig. 5a). A microchannel system is shown, and the left half of it is filled with blue dye, while the nanoparticle chains are located at the right chamber. At *t* = 20 s, the RPNS is generated and moves towards the microchannel, and due to the flow induced at its tip, the blue dye is pushed back into the left chamber, as shown at 40 s. Then we wait for ~140 s, the blue dye will not diffuse into the right chamber or even the microchannel again. Another experiment with free diffusion of the blue dye is shown as the control. Moreover, an RPNS may also be applied as a sorting device, as shown in Fig. 5b. First, three 70 μm-diameter beads are mixed with hundreds of 15 μm-diameter beads in a fluidic channel, as shown at *t* = 0 s. An RPNS is formed and with magnetic navigation, it moves towards the microbeads. Due to the strong fluidic flow generated at the tips, the 70 μm-diameter beads are trapped by the RPNS and move with the swarm body. In contrast, the small beads (15 μm) act differently, that one part of them are directly push aside by the flow (as shown by the green arrows), the other part is attracted into the swarm, and then they are pumped out at some locations near the swarm tip (the same phenomenon with Supplementary Fig. 16). As a result, small particles are pushed away sideways while large particles are trapped in front of the ribbon-like microswarm. Finally, as shown in Fig. 5b (*t* = 150 s), when the RPNS passes through the microchannel, only the selected large particles are sorted out, and small particles are left inside the channel.

## Discussion

We present a strategy that is capable of triggering paramagnetic nanoparticles into a ribbon-like dynamic microswarm with a high pattern stability, using programmed oscillating magnetic fields. The mobile microswarm is capable of performing reversible anisotropic deformation with its aspect ratio changing over one order of magnitude, as well as the controlled splitting and merging. The technique provides insights into the generation, reconfiguration and navigated locomotion of a microswarm. Our work provides support to understand the swarm behaviour at small scales and the corresponding control methods from several aspects. The generation and the pattern control of the microswarm significantly depend on the particle–particle interaction, which may pave the way for understanding the complex morphological transformations of living systems. The navigated locomotion and the pattern stability are also investigated in highly confined environments with varied boundary conditions. The microswarm may provide inspiration and solution for applications such as targeted delivery, maskless ribbon-like patterning for microfabrication and micromanipulation.

## Methods

### Materials and preparation for the experiments

In our experiments, magnetite nanoparticles with a diameter of 500 nm were prepared (Supplementary Fig. 17) using solvothermal method, which has been previously reported^28^. For magnetic actuation, first, the synthesised nanoparticles are subjected to an ultrasonic bath for 5 min. One drop of the nanoparticle solution (8 μL, 0.6 wt%) is then added into the tank, which is filled with deionised (DI) water (~1.5 mL). Based on our experimental methods, the minimum weight of the paramagnetic nanoparticles that each experiment requires is 10 μg, in order to generate a dynamically stable RPNS. Then the diffused particles are gathered by placing a permanent magnet under the tank. The magnet is slightly moved in a small region for a better gathering of the particles. Then, the nanoparticle clusters are transferred into the workspace of the electromagnetic setup. In the experiments, a piece of silicon wafer is used as the substrate and with its polished surface upwards, to reduce the adhesion of nanoparticles and enhance the observation contrast. In order to create a uniform distribution of the nanoparticle chains, a reported dynamic magnetic field with a frequency of 20 Hz is applied to disassemble the particle clusters while spreading them^29^. After the disassembly process, the nanoparticles are ready for further magnetic actuation processes.

### Electromagnetic actuation platform

The magnetic actuation and control experiments are conducted in a 3-axis Helmholtz electromagnetic coil setup (Supplementary Fig. 18). The control signals are generated by a PC, and then the current is input into the coils to generate magnetic fields in the working space. We are able to use the setup to generate oscillating magnetic fields with specific requirements, by inputting mathematical expressions into the control program.

### Data availability

All the relevant data used to prepare this manuscript and the Supplementary Information is available upon request.

## Electronic supplementary material

Supplementary Information

Description of Additional Supplementary Files

Supplementary Movie 1

Supplementary Movie 2

Supplementary Movie 3

Supplementary Movie 4

Supplementary Movie 5

Supplementary Movie 6

Supplementary Movie 7

Supplementary Movie 8

Supplementary Movie 9

Supplementary Movie 10
