# Supplementary material for: Ultra-extensible ribbon-like magnetic microswarm
Source: Nat Commun. 2018 Aug 21;9:3260. doi: 10.1038/s41467-018-05749-6 (PMC6104072; doi:10.1038/s41467-018-05749-6)
Supplement: Supplementary file 1 — Supplementary Information [file 41467_2018_5749_MOESM1_ESM.pdf]

Supplementary Information

Ultra-extensible Ribbon-like Magnetic Microswarm

Jiangfan Yu[1], Ben Wang[1], Xingzhou Du [1], Qianqian Wang[1],and Li Zhang[1,2,3*]

[1]Department of Mechanical and Automation Engineering, The Chinese University of Hong Kong, Shatin, N.T., Hong Kong SAR, China.

[2]Chow Yuk Ho Technology Centre for Innovative Medicine, The Chinese University of Hong Kong, Shatin, N.T., Hong Kong, China.

[3]T-Stone Robotics Institute, The Chinese University of Hong Kong, Shatin, N.T., Hong Kong, China.

*To whom correspondence should be addressed. E-mail: lizhang@mae.cuhk.edu.hk

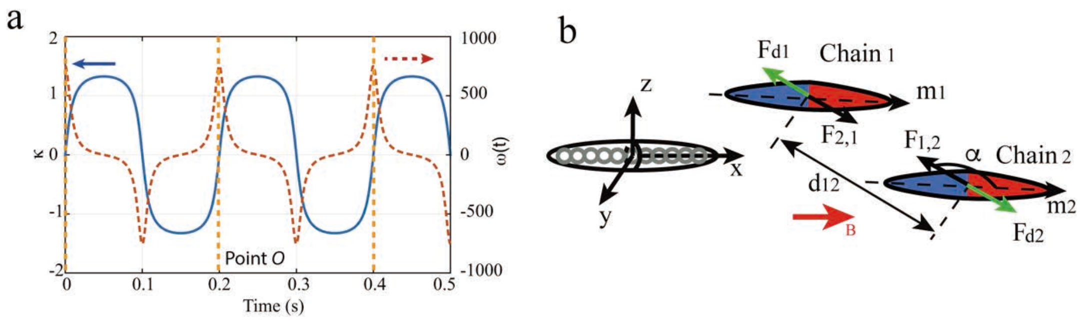

Supplementary Figure 1: a, The changes of the oscillation angle $\kappa(t)$ and the angular velocity ω(t) with time. The blue line indicates the change of the oscillation angle $\kappa(t)$, while the red dashed line indicates the change of the angular velocity $\omega(t)$. **b**, The coordinate system and the prolate spheroid simplified from a particle chain. The magnetic interaction forces are $F_{2,1}$ and $F_{1,2}$, the distance between the two spheroids are d_{12}, and the two spheroids have magnetic dipole moments of m_1 and m_2, respectively.

Supplementary Note 1: Disassembly behaviour of a particle chain: The oscillating angle $\kappa(t)$ and the angular velocity ω(t) of the oscillating field depicted in Fig. 1a can be expressed as:

$$\tan\kappa(t) \;= \gamma\sin(2\pi f t) \tag{1}$$

$$\omega(t) = \frac{d}{dt}\kappa(t) = \frac{2\pi\gamma f\cos(2\pi f t)}{1+\gamma^2\sin^2(2\pi f t)}, \tag{2}$$

and the curves are plotted in Supplementary Fig. 1a. The blue curve and the red dashed curve represent the changes of $\kappa(t)$ and $\omega(t)$ with time, respectively. Both the curves are periodic, and the angular velocity is significantly nonlinear. The particle chain oscillates much faster at the middle point [see Point O in Fig. 1a] than at the two ends of a cycle motion.

Actuated by magnetic fields with different strengths, the paramagnetic nanoparticles form chains with different lengths. In order to estimate the relationship between the chain lengths and the applied field strength, a modified Mason Number R_T [1] can be used, and combining

$\boldsymbol{B} = \boldsymbol{B}_{\mathbf{AC}} + \boldsymbol{B}_{\mathbf{cons}}$, where $\boldsymbol{B}$ is the superposed magnetic field, $\boldsymbol{B}_{\mathbf{AC}}$ is the sinusoidal alternating magnetic field and $\boldsymbol{B}_{\mathbf{cons}}$ is the constant magnetic field (please refer to Figure 1). The relationship can be expressed as:

$$64\frac{\mu_0\eta\omega}{\chi_p^2}\frac{N^2}{(ln(N)+\frac{1.2}{N})} - A^2sin^2(2\pi ft) - C^2 = 0 \quad (3)$$

where χ_p and η are the effective magnetic susceptibility constant of the nanoparticles, and the dynamic viscosity of the fluid, respectively. The chain consists of (2N+1) particles. In order to investigate the relationship between field strength and chain length, the angular velocity here is regarded to be constant.

Supplementary Note 2: Magnetic chain-chain interaction: Magnetic interaction is an important factor for the generation of an RPNS. In this work, we regard the nanoparticle chains as the minimum components of the microswarm, which are simplified to be prolate spheroids. The chain-chain magnetic interactions are first investigated, and the schematic drawing is shown in Supplementary Fig. 1b. Two magnetic spheroids exert attractive forces on each other, indicated by $F_{1,2}$ and $F_{2,1}$. During the locomotion, fluidic drags, F_{d1} and F_{d2} are exerted in the opposite direction of the motion.

When the external magnetic field is smaller than the saturation magnetization of the particle chains, the magnetisation of the chains can be expressed as [2]:

$$\boldsymbol{m} = \frac{V_c\chi_\alpha}{\mu_0(1+\chi)}\boldsymbol{B}\,, \quad (4)$$

where $\mu = \mu_0(1+\chi)$ is the permeability of the particle chains, χ is the magnetic susceptibility constant, and χ_α is the apparent susceptibility tensor that is related to the shape

of the chains. Herein, the chains are considered as axisymmetric prolate spheroids, as shown in Fig. S1b. In this case, the susceptibility tensor is expressed as [3]:

$$\chi_a = \begin{pmatrix} \frac{\chi}{(1+n_a\chi)} & 0 & 0 \\ 0 & \frac{\chi}{(1+n_b\chi)} & 0 \\ 0 & 0 & \frac{\chi}{(1+n_b\chi)} \end{pmatrix}, \quad (5)$$

where n_a and n_b are demagnetising factors along the major (x-axis) and minor axes (y-axis and z-axis), given by $n_a + 2n_b = 1$ and $n_a = \frac{1-\varepsilon^2}{2\varepsilon^3}(log(\frac{1+\varepsilon}{1-\varepsilon}) - 2\varepsilon)$, and $\varepsilon = \sqrt{1-\Lambda^2}$ is the eccentricity. $\Lambda = a/b$ is the aspect ratio of the prolate spheroid. Therefore, the magnetic dipole moments of the chains can be expressed as:

$$\begin{pmatrix} m_x \\ m_y \\ m_z \end{pmatrix} = \frac{V_m\chi}{\mu_0(1+\chi)} \begin{pmatrix} \frac{B_x}{1+n_a\chi} \\ \frac{B_y}{1+n_b\chi} \\ \frac{B_z}{1+n_b\chi} \end{pmatrix}. \quad (6)$$

The magnetic field induced at point p, by the ith paramagnetic chain located at $p_i = (x_i, y_i, z_i)^T$ with its dipole moment m_i, is given by [4]:

$$B_{pi}(p) = \frac{\mu_0}{4\pi}(3(m_i \cdot n_i) - m_i) \quad (7)$$

where $\mu_0 = 4\pi \times 10^{-7}$ Tm/A is the vacuum permeability, and $n_i = \frac{p-p_i}{||p-p_i||}$ is the separation distance unit vector. The interaction forces exerted on the (i+1)th chain by the ith chain are expressed as:

$$\boldsymbol{F}_{i,i+1} = -\frac{\mu_0}{4\pi}\nabla((\nabla\frac{\boldsymbol{m}_i \cdot \boldsymbol{d}_{i,i+1}}{d_{i,i+1}^3}) \cdot \boldsymbol{m}_{i+1}), \quad (8)$$

where $\boldsymbol{d}_{i,i+1} = d_{i,i+1}\boldsymbol{n}_i$ is the separation distance vector between the two chains, as illustrated in Fig. S1b. Therefore, an increasing external magnetic field strength can

significantly enhance the interaction forces between two chains.

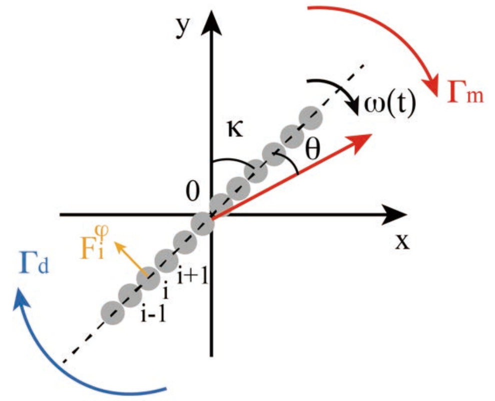

Supplementary Figure 2: The schematic drawing of a particle chain oscillating with the applied magnetic field. The oscillating angle is κ, the phase lag is θ, and the angular velocity at the moment is ω(t). The force exerted on the ith particle by the other particles, perpendicular to the long axis of the chain, is F_{φ}. Magnetic torque Γ_m and drag torque Γ_d exert oppositely on the chain during the oscillation.

Supplementary Note 3: Oscillating behaviours of a particle chain: Actuated by the oscillating magnetic field, the paramagnetic nanoparticles are first assembled into chain-like structures due to the dipole-dipole attractive interactions. Then, the nanoparticle chains oscillate with the input oscillating magnetic field, as shown in Supplementary Fig. 2. The applied field is shown in Fig. 1a. During the oscillating motion, the actuating magnetic torque Γ_m and the resistive fluidic drag torque Γ_d are exerted on a particle chain, which are represented by the red and blue arrows, respectively. The angular velocity of the chain $\omega(t)$ is time-dependent, and the angle between the chain and y-axis at the moment is κ. A phase-lag angle, between the input field and the particle chain, is shown by θ. Herein, we investigate the magnetic interaction between particles using a single uniform particle chain model.

Consider a chain formed by (2N+1) particles, the middle particle is located at the origin of the coordinate, and the remaining particles are labelled from -N to N. The radius of the particles is a. Because the Reynolds number in this case is estimated to be approximately 0.006, the inertia term during the motion of the particle chains can be neglected. Therefore, Γ_m and Γ_d are always counterbalanced during the oscillation, and the two torques can be expressed as [2]：

$$\Gamma_m = 2\sum_{i=1}^{N}(F_i^{\varphi} r_i) = \frac{3\mu_0 m^2}{4\pi} sin\,(2\theta) \sum_{i=1}^{N}(2r_i \sum_{\substack{j=-N \\ i\neq j}}^{N} \frac{1}{r_{ij}^4}) \tag{9}$$

$$\Gamma_d = \frac{64\pi a^3}{3} \frac{N^3}{ln(N)+\frac{1.2}{N}} \eta\omega(t) \tag{10}$$

where F_i^{φ} is the force exerted on the ith particle, by the other $2N$ particles, and the force is perpendicular to the long axis of the chain. The distance between the ith particle and the centre of the chain is r_i. The permeability of the free space and the magnetic dipole moment of the paramagnetic nanoparticles, are represented by μ_0 and m, respectively. The centres of the ith and j^{th} particles have a distance of r_{ij}. The dynamic viscosity of the fluid, in which the experiments are conducted, is indicated by η.

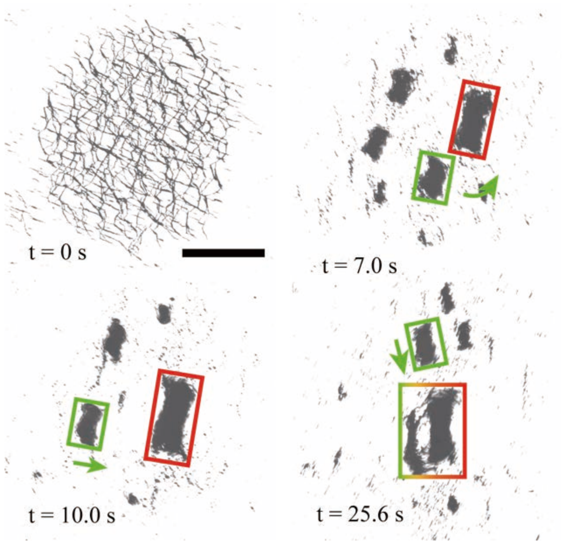

Supplementary Figure 3: Self-merging processes during the generation of the final RPNS. The scale bar is 800 μm.

Supplementary Note 4: Self-merging during the generation process: When the oscillating magnetic field is applied, the nanoparticles immediately locally gathered into dynamic ribbon-like subswarms with a high particle concentration (Supplementary Fig. 3). The fluidic interactions among the subswarms are significant, and a series of self-merging processes are performed. Gradually, a major subswarm that governs the flow field will be generated (labelled by the red rectangles) and exerts strong fluidic attractive interactions on smaller ones (labelled by the green rectangles). The smaller subswarms gradually approach and finally merge into the major one. A head-to-tail merging occurs at 7.0 s. We also observe a side-by-side self-merging between two subswarms (10.0 s - 25.6 s). The smaller subswarm is attracted by the major one laterally and then, it is deformed, split apart, and attracted into the two ends of the major subswarm.

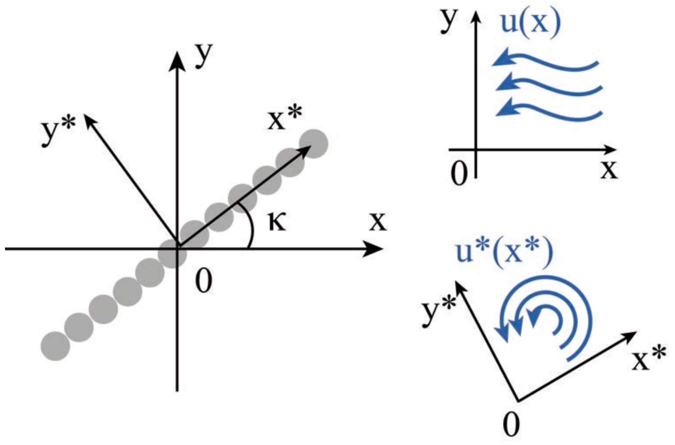

Supplementary Figure 4: The world frame $x{-}o{-}y$ and the body frame $x^{*}{-}o{-}y^{*}$. The flow $u(x)$ is passive in the world frame, and in the body frame, the chain keeps statically embedded in a oscillating background flow $u^{*}(x^{*})$.

Supplementary Note 5: Fluidic velocity field induced by a single oscillating chain: The governing equations for an incompressible fluid system are given by the Navier-Stokes equations, as:

$$\frac{\partial \boldsymbol{u}}{\partial t} + \boldsymbol{u} \cdot \nabla \boldsymbol{u} = -\frac{1}{\rho} \nabla p + \nu \nabla^2 \boldsymbol{u} \tag{11}$$

$$\nabla \cdot \boldsymbol{u} = 0 \tag{12}$$

where ρ is the fluid density, $\boldsymbol{u}$ is the velocity field, p is the pressure, and ν is the kinematic viscosity of the fluid. The solution for the velocity field of the fluid, induced by an oscillating particle chain is investigated, through linear transformations of the body frame solutions built by [5]. The particle chains are simplified to prolate spheroids.

As shown in Supplementary Fig. 4, the coordinate x–o–y is the world frame, and x^*-o-y^* is the body frame. In the world frame, the spheroid oscillates, and the background flow remains passive. In the body frame, the spheroid is statically embedded in the oscillating background flow. In the body frame, the equation of the spheroid is shown as:

$$\frac{{x^*}^2}{a^2} + \frac{{y^*}^2 + {z^*}^2}{b^2} = 1 \quad (a > b) \tag{13}$$

The slenderness of the spheroid is defined as $\delta = b/a$, and the eccentricity is $e = \sqrt{1 - \delta^2}$. The oscillation of the x*-o-y* plane is represented by $U(x^*) = -\omega(t)(e_z \times x^*)$, and e_Z is oriented in the positive z direction. The transformations are defined by the matrix:

$$R_\kappa = \begin{pmatrix} \sin\kappa(t) & 0 & -\cos\kappa(t) \\ 0 & 1 & 0 \\ \cos\kappa(t) & 0 & \sin\kappa(t) \end{pmatrix} \tag{14}$$

where R_κ is a clockwise rotation transformation matrix and, $\kappa(t)$ is the time-dependent oscillating angle. The velocity field $\boldsymbol{u}(x)$ is constructed in the body frame, as:

$$u^*({x_0}^*) = 0 \tag{15}$$

$$\lim_{x\to\infty} u^*(x^*) = R_\kappa^T U(R_\kappa x^*) = \omega \begin{pmatrix} y\sin\kappa(t) \\ -x\sin\kappa(t) + z\cos\kappa(t) \\ -y\cos\kappa(t) \end{pmatrix} \tag{16}$$

where x_0 lies on the surface of the spheroid defined in Equation 13. Therefore, in the world frame, the transformation and the velocity field in the world frame can be expressed as:

$$\boldsymbol{x} = R_\kappa \boldsymbol{x}^* \tag{17}$$

$$\boldsymbol{u}(\boldsymbol{x}) = -\boldsymbol{U}(x) + R_\kappa \boldsymbol{u}^*(R_\kappa^T \boldsymbol{x}) \tag{18}$$

In world frame, the construction of the solution that satisfies the boundary conditions is:

$$\boldsymbol{u}(\boldsymbol{x_0}) = -\boldsymbol{U}(x) \tag{19}$$

$$\lim_{x\to\infty} \boldsymbol{u}(\boldsymbol{x}) = 0. \tag{20}$$

The boundary conditions in Equations 15 and 16 coupled with the Stokes equations describes the velocity field $\boldsymbol{u}(x)$ due to the oscillating motion of a spheroid, with the oscillation angle of $\kappa(t)$. From Equation 17, the velocity field can be constructed as the sum of four contributions, due to the linearity of the Stokes equations [6]:

$$\boldsymbol{u}(\boldsymbol{x}) = \boldsymbol{u}^1(\boldsymbol{x}) + \boldsymbol{u}^2(\boldsymbol{x}) + \boldsymbol{u}^3(\boldsymbol{x}) + \boldsymbol{u}^4(\boldsymbol{x}) \tag{21}$$

where $u^i(x_0) = 0$ is for i=1, 2, 3, and 4, while $\lim_{x\to\infty} \boldsymbol{u^1}(\boldsymbol{x}) = \omega y\sin\kappa(t)$, $\lim_{x\to\infty} \boldsymbol{u^2}(\boldsymbol{x}) = -\omega x\sin\kappa(t)$, $\lim_{x\to\infty} \boldsymbol{u^3}(\boldsymbol{x}) = \omega z\cos\kappa(t)$, and $\lim_{x\to\infty} \boldsymbol{u^4}(\boldsymbol{x}) = -\omega y\cos\kappa(t)$.

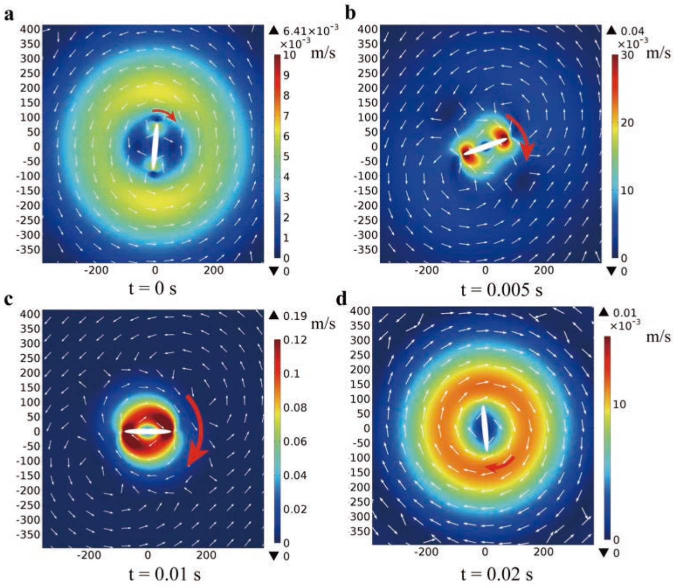

Supplementary Figure 5: The simulation results for the flow field induced by an oscillating spheroid at (a) $t = 0$ s, (b) $t = 0.005$ s, (c) $t = 0.01$ s, and $t = 0.02$ s.

The fluidic velocity field induced by a single oscillating spheroid (simplified from a particle chain) is simulated, as shown in Supplementary Fig. 5. The oscillating direction is labelled by the red arrows, and the size of the arrows indicates the magnitude of the angular velocity of the spheroid. Because of the variable angular velocity of the chains, the velocity of the chains increases significantly from 0 s to 0.01 s. The fluid velocity field becomes stronger and after 0.01 s, becomes weak with a decrease in the angular velocity of the spheroid. When the chain just changes the direction of oscillation (Supplementary Fig. 5, 0.005 s), vortices will be induced at the ends of the chain.

Supplementary Note 6: Particle configuration formed at the tips of the swarm:

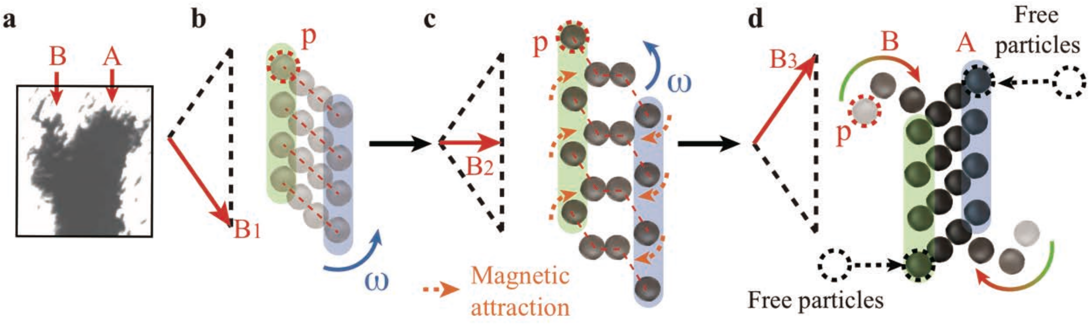

Supplementary Figure 6: Schematics of the dynamic configurations of the nanoparticles formed at tips of an microswarm with the actuation using the oscillating field.

At the tips of the swarm, the behavior of the nanoparticles is different from those in the middle part (refer to Supplementary Fig. 6a). Initially, the swarm is regarded as several nanoparticle chains arrayed in a ribbon-like pattern, and the magnetic field B1 is shown by the red arrow (Supplementary Fig. 6b). The left and right rows of nanoparticles are labeled with the green and blue areas, respectively. The red dotted lines link each particle in a chain, showing the initial configuration of the nanoparticles. The magnetic field oscillates with decreasing field strength (Supplementary Fig. 6c), and long chains cannot be maintained. The left and right nanoparticles are disassembled from the main body, and reconfiguration occurs due to the magnetic attraction. The disassembled nanoparticles are attracted by the adjacent particle chains, as shown by the orange dotted arrows in Supplementary Fig. 6c. Particle p is firstly dominated by fluidic repulsive forces, and then the magnetic dipole forces attract it (Supplementary Fig. 6c and d). Therefore, a moving trajectory of particle p is generated as shown by the curves in Supplementary Fig. 6d. The green parts of the curves illustrate that the repulsive fluidic forces are governing, while the red parts demonstrate that the magnetic

attraction forces become the main factor. Therefore, in part A, the particle configuration is solid due to particle-particle magnetic interactions; and in part B, the particle configuration is loose, because some particle clusters are encountering the reconfiguration process. As a conclusion, at the tips of a swarm, disassembled clusters have the space to be firstly repulsed by fluidic interaction, and then attracted by magnetic interactions; while in the middle part of the swarm, small clusters after disassembly will be attracted by other adjacent particle chains immediately.

If the entire swarm has a translational velocity, as shown in Supplementary Fig. 7, the yellow arrow indicates the direction of the velocity **v**. In this case, the particle **p** will be firstly repulsed by the fluidic flow, however, due to the microswarm has a velocity **v**, the distance between the main body of the swarm and particle **p** is enlarged. The magnetic dipole interaction between them is decreased, which hinders the attraction of particle **p**. Therefore, if **v** is too large, particle **p** may not be attracted towards the main swarm again, and it will be lost during translation. In order to avoid this situation, to lower the translational velocity is a good option, which enables the particles in the reconfiguration processes to be attracted into the swarm again.

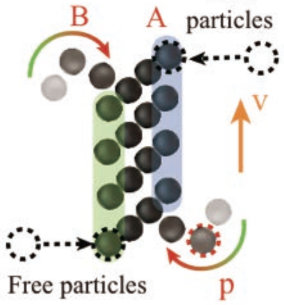

Supplementary Figure 7: The schematics when an RPNS has a translational velocity.

Supplementary Note 7: Microswarm generated by smaller particles (diameter: 100 nm and 250 nm):

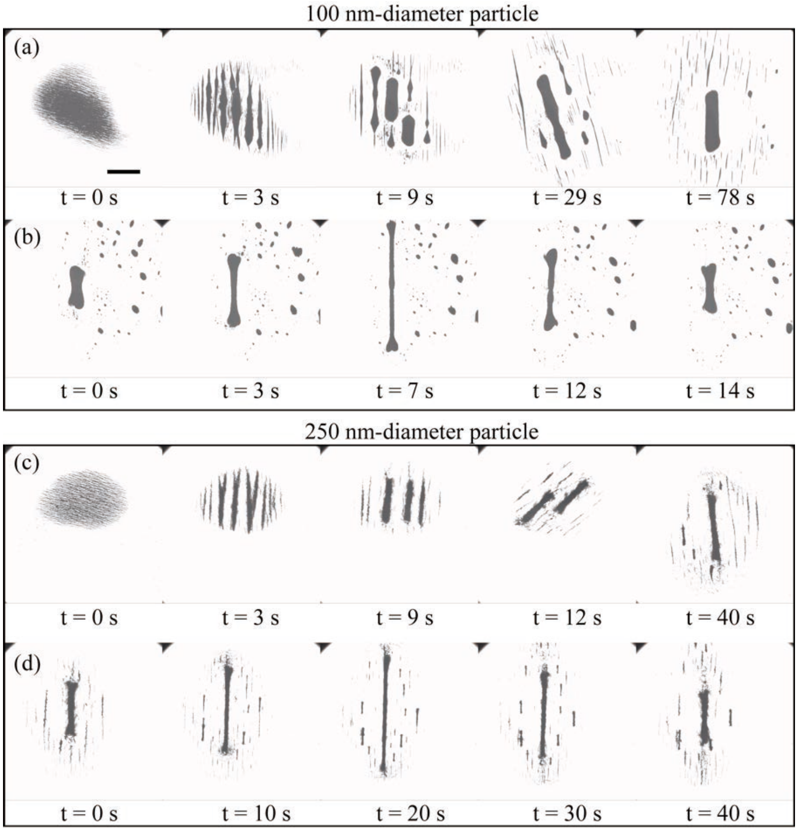

Supplementary Figure 8: RPNS generation and reconfiguration using 100 nm-diamter particles and 250 nm-diameter particles.

We use two more kinds of nanoparticles, with a diameter of 100 nm and 250 nm, to realize the experiments, as shown in Supplementary Fig. 8. For 100 nm-diameter particles, the generation takes more time than larger-particle cases, because the decrease of the particle volume leads to the decrease of the magnetic dipole interactions. The elongation and shrinkage of the swarm in both cases are successful, indicating that when the diameter of the particles is downscaled to 100 nm, our assembly technique remains effective.

Supplementary Note 8: The generation process with different initial particle concentrations:

Hereby, we give two additional cases when the initial particle concentrations are 2.1 $\mu g/mm^2$ and 6.5 $\mu g/mm^2$, respectively, as shown in Supplementary Fig. 9. When the particle areal concentration is low, more small subswarms will be generated during the process, and the distances among them are relatively large, which hinders the self-merging among subswarms. Meanwhile, when higher areal concentration of nanoparticles is applied, fewer subswarms with larger size will be generated initially, and they are relatively close to each other. In this case, the subswarms have a higher chance to merge into larger ones.

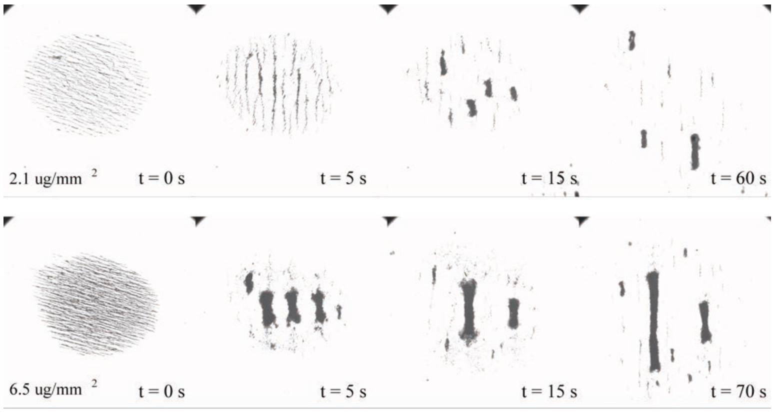

Supplementary Figure 9: The generation process of an RPNS when the initial particle densities are 2.1 $\mu g/mm^2$ and 6.5 $\mu g/mm^2$. The areal density of the nanoparticles for the experiments in the manuscript is approximately 4 $\mu g/mm^2$.

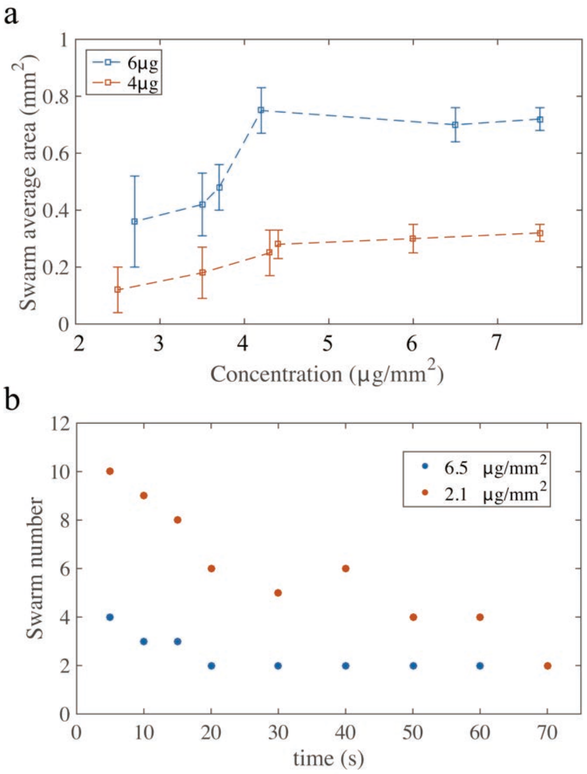

Supplementary Figure 10: (a) The relationship between the particle concentration and the swarm size. (b) The change of swarm number with time.

Moreover, we made characterizations about the relationships among particle concentration, subswarm size and the number of subswarms, as shown in Supplementary Fig. 10. As shown in Supplementary Fig. 10 (a), with the increase of the particle concentration, the average area of a stable RPNS increases. Compared with the blue and red dashed curves, an RPNS will have a larger area when more nanoparticles are used. Moreover, when the particle concentration is low, more sub-swarms will be formed during the generation of an RPNS, as shown in Figure Supplementary Fig. 10 (b). In both cases, the number of the subswarms gradually decreases due to the self-merging behaviors.

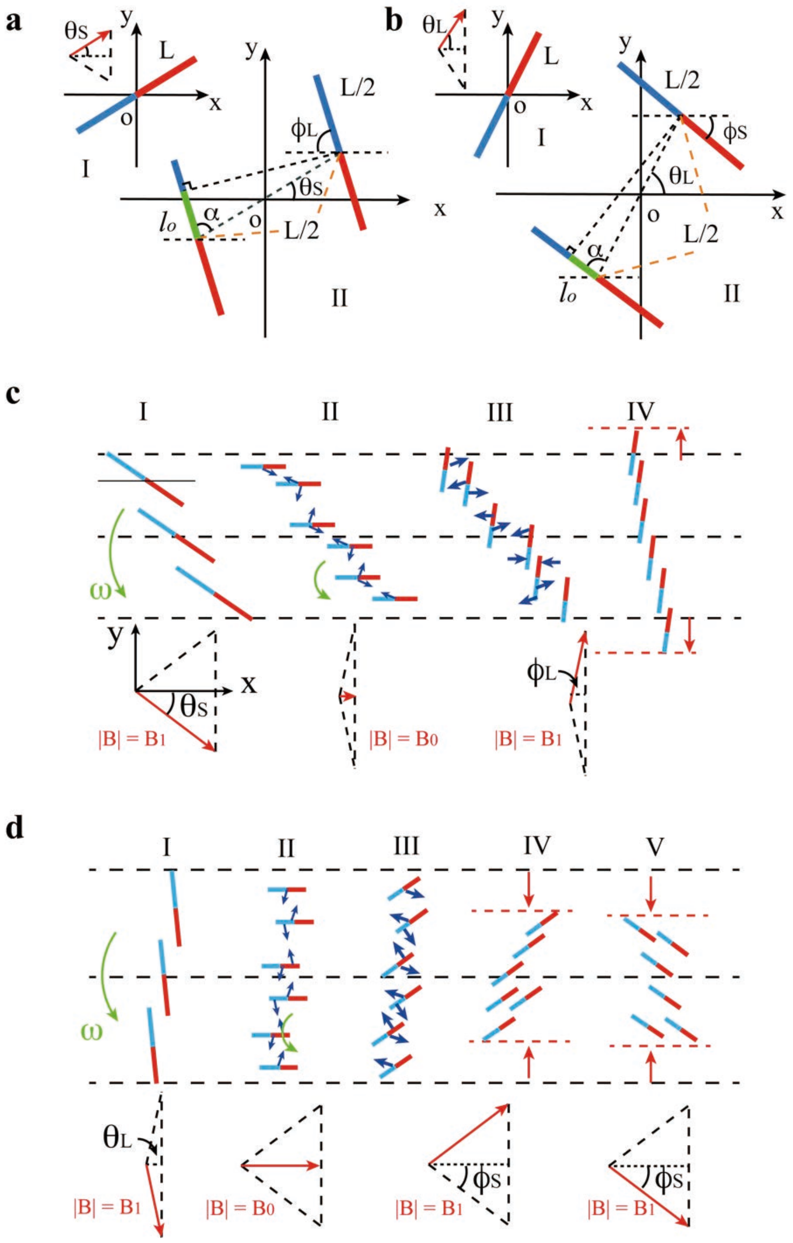

Supplementary Figure 11: a-b, The relative positions and different forces exerted on two magnetic particle chains. **a,** From a small oscillating angle θ_S to a large one ϕ_L. **b**, From a large oscillating angle θ_S to a large one ϕ_L. **c**, The schematic process of the elongation process of an RPNS. **d**, The schematic process of the shrinkage process of an RPNS.

Supplementary Note 9: Chain-chain magnetic interaction and the schematic mechanisms of the elongation and shrinking behaviors: The induced interaction forces between neighbouring particle chains, with different relative positions, are investigated as shown in Supplementary Fig. 11a,b. The relative positions of the chains can be different by tuning their oscillation angles. Two cases are analysed, which are from a small initial oscillation angle θ_s to a large one φ_L, and from a large initial oscillation angle θ_L to a small

one φ_S, respectively. The red and blue parts indicate the magnetised dipoles of the particle chains. The chain actuated by the fields with a small initial oscillation angle θ_S is shown in stage I (Supplementary Fig. 11a). In stage II, the two chains are split from the chain in stage I, and the positions of their centres remain unchanges. The original and current oscillation angles (i.e., θ_S and φ_L) are labelled. The length of the green line $l_O = \frac{L}{2}\cos\alpha$, which directly shows the overlap of the same dipoles, is critical. If $l_O < L/4$, the overlap exists between the same dipoles (the blue part), and repulsive forces are induced; if $l_O > L/4$, attractive interactions are induced. In (b), the mechanisms are similar.

In these two cases, α can be expressed as:

$$\alpha = \pi - (\theta + \phi) \tag{22}$$

where θ can be θ_S or θ_L, and ϕ can be ϕ_L or ϕ_S, respectively. As a result, when $\theta + \varphi > 120^\circ$, attraction forces are induced, while when $\theta + \varphi < 120^\circ$, the induced forces are repulsive at this moment. In fact, attractive and repulsive forces play similar roles in the reconfiguration of particle chains. Attractive forces lead to smaller chain-chain distances to form a concentrated pattern with a striped shape. Meanwhile, repulsive forces will not always enlarge the chain-chain distances, but actuate the chains to be staggered along the direction of the magnetic fields, until the overlap of the same dipoles disappears. Then, the interaction forces become attractive, which also leads to the reconfiguration of the swarm pattern.

The mechanisms for the elongation of an RPNS are schematically shown in Supplementary Fig. 11c and Supplementary Movie 6. In stage **I**, the particle chains oscillate with a small oscillation angle θ_S. When the magnetic field strength is reduced to the minimum value, i.e., in stage **II**, the chains split into shorter ones for reconfiguration, and in this stage, the location of each chain is maintained. From stage **II**, the oscillation angle is increased to φ_L. Attractive

forces produce smaller smaller chain-chain distances to form a concentrated pattern with a ribbon-like shape. Meanwhile, the repulsive forces will not always enlarge the chain-chain distances. Because the chains tend to reach the tip of the adjacent chains with different dipoles, they are actuated to be staggered along the direction of the magnetic fields due to magnetic repulsive forces, until the overlap of the same dipoles disappears. Then, the interaction forces become attractive, which also leads to a reconfiguration of the swarm pattern. Due to the small initial oscillation angle θ_S, the chains have a relatively wide distribution along the x-axis (stage **II**). When the chains reach the full oscillation angle φ_L of the applied field with increased γ, as shown in stage **III**, the overlaps of the same dipole among particle chains appear. Due to the induced repulsive forces, the adjacent chains tend to reach the tips with different dipoles of each other along the direction of the magnetic field. As a result, the overlaps among particle chains are reduced, leading to the significant elongation of the microswarm pattern along the y-axis, whereas the width of the pattern is decreased because of the attractive forces along the x-axis (stage **III** and **IV**).

The shrinking behavior of an RPNS is shown in Supplementary Fig. 11d, the original oscillation angle θ_L is large, as shown in stage I. An oscillating field with a smaller oscillation amplitude is applied in stage II. Then, the particle chains split, and in this stage, the chains have no locomotion. In stage III, when the chains reach the full oscillation amplitude, the overlaps of the particle chains decrease due to the magnetic repulsive forces along the direction of the magnetic field, with the same mechanism in Supplementary Fig. 11c. However, because of the small oscillation angle φ_S and the initially wide distribution along the y-axis, when the attractive forces pull the chains towards each other, the length of the

swarm along the y-axis is shrunk, as shown in stage IV.

Supplementary Note 10: Phase diagram for controlled splitting:

The phase diagram is presented in Supplementary Fig. 12. Splitting an RPNS into 2, 3, 4, 5 subswarms can be stably realized, while if more subswarms are required, different results will appear with the same oscillating magnetic field. For example, when the swarm rotates with a frequency of 0.35 Hz, and the amplitude ratio is 4, an RPNS will split into 5 or 6 subswarms.

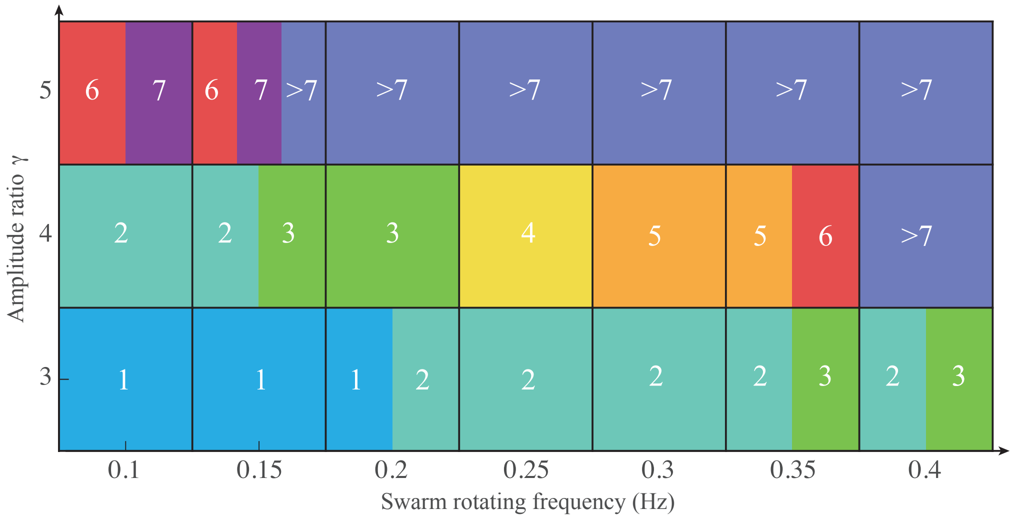

Supplementary Figure 12: The phase diagram showing the relationship among the swarm rotating frequency, amplitude ratio and the number of the split subswarms. The white number shows the number of the spit subswarms. The data is obtained from five independent experiments.

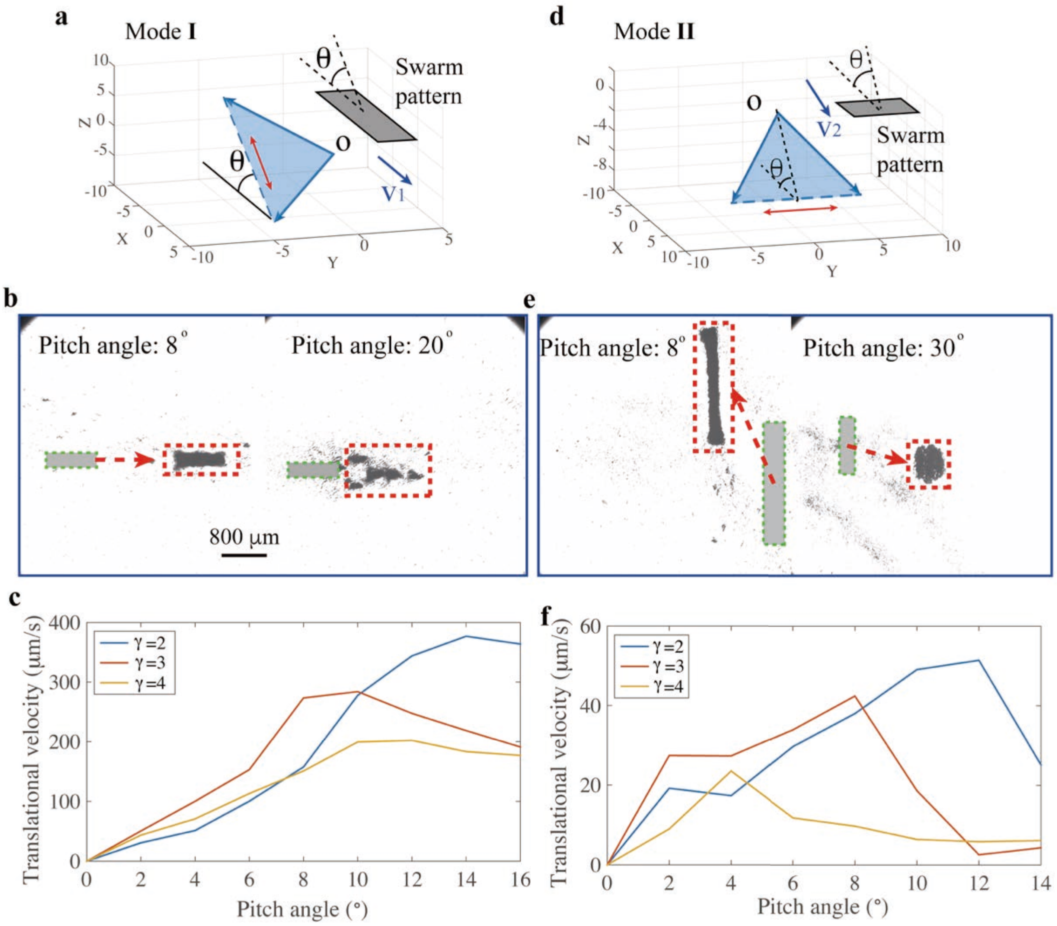

Supplementary Figure 13: a, The schematic demonstration of the applied oscillating field to actuate an RPNS along its long sides (forward). The pitch angle is represented using θ. **b**, The swarm morphologies during forward locomotion with pitch angles of 8 and 20. In this case, g = 3, the field strength is 10 mT, and the oscillating frequency is 30 Hz. **c**, The velocities of forward locomotion with different pitch angles, when the oscillating frequency is 30 Hz. The schematic depiction of the applied field, the morphology of the swarm with different pitch angles, and the translational velocities when an RPNS moves along its short side (laterally), are shown in **d-f**, respectively. The dotted green rectangles show the position and pattern of the initial RPNSs. Each data point represents the average of 3 experiments. The error bar indicates the standard deviation (s.d.). The scale bar is 800 μm.

Supplementary Note 11: Motion characterization of an RPNS: RPNSs are capable of performing forward, backward and lateral locomotion by modifying the applied magnetic field, which significantly improves its motion flexibility in confined environments. In mode I, if the magnetic field oscillates along the trajectory tilted to the experimental plane

(Supplementary Fig. 13a, blue dashed line and red arrows), the RPNS will move forward (Supplementary Fig. 13c, blue arrow). In mode II, if the trajectory is parallel to the experimental plane (Supplementary Fig. 13b, blue dashed line and red arrows), the swarm moves laterally with significant drifting (Fig. 4d, left image). Moreover, increasing the pitch angle of the oscillating field not only modifies the translational velocity of an RPNS, but also influences its dynamic pattern considerably. Actuated by the magnetic field in mode I with a small pitch angle (e.g., less than 10°), all the particles move as an entity, and the pattern of the swarm stays intact during the motion (Supplementary Fig. 13b, left image). However, the swarm is unstable during locomotion at a larger pitch angle, and the pattern is uncontrollably split into several parts (Supplementary Fig. 13b, right image). The experimental results when the RPNS is actuated by the field in mode II with a small pitch angle is shown (Supplementary Fig. 13e, left image). When the applied pitch angle is increased (i.e., 30°), the RPNS further shrinks out of control and becomes unstable during the motion (Supplementary Fig. 13e, right image). Therefore, the RPNSs are stable with good controllability when the pitch angle is small (i.e., normally less than 10°).

The moving velocities in mode I and II are measured and analyzed. In mode I, when the oscillating frequency is 30 Hz, the relationship between the translational velocity and applied pitch angle, with different amplitude ratios is shown (Supplementary Fig. 13c). The velocities first increase with the pitch angle, and when the pitch angles exceed a specific value (e.g., 10° when $g = 3$), the velocities gradually decrease. When the oscillating frequency is maintained, the maximum velocity that the microswarm can reach has an apparent relationship with the amplitude ratios, namely, that increasing the amplitude ratio reduces the maximum translational velocity. In mode II, similar regularities can be observed (Supplementary Fig. 13f); however, after the velocity reaches the maximal value, the decrease is much more significant.

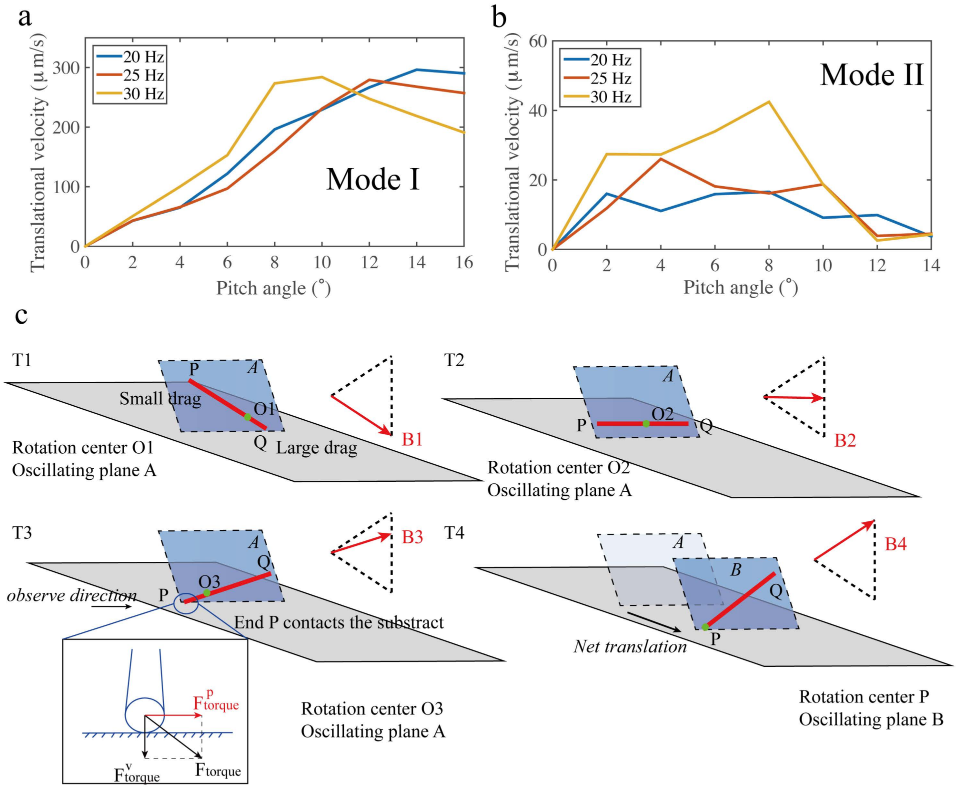

Supplementary Figure 14: a, The velocities of forward locomotion with different pitch angles, when the oscillation ratio is 3. **b,** The velocities of lateral locomotion with different pitch angles, when the oscillation ratio is 3. **c**, The schematics for a nanoparticle chain making translational locomotion actuated by the oscillating magnetic field. The red rods indicate magnetic nanoparticle chains, while the blue surfaces show the oscillating planes. The grey surfaces indicate the substrates. The input magnetic fields are shown at each moment.

The relationships between the translational velocity and the pitch angles in the two modes (Supplementary Fig. 13a,d) are shown in Supplementary Fig. 14a,b. The schematic explanation of the translational locomotion of the swarm is shown in Supplementary Fig. 14c. It is known that near a solid boundary, introducing a pitch angle into the actuating magnetic field can make the fluidic drag encountered by upper and lower parts of the object different, and the corresponding moving velocities are different as well. Now we focus on mode I in Supplementary Fig. 13a, the schematic demonstration of the translational locomotion of a particle chain PQ (the red rod in Supplementary Fig. 14c) is presented in Supplementary Fig. 14c. First, due to the different drag encountered by the upper and down parts of the chain PQ, the oscillating center of the chain is located at O1, which is at the lower part of PQ, as shown in T1. Then the geometry center of PQ becomes closer to the substrate at T2. The chain is capable of following the actuating oscillating field, until end P contacts the substrate at T3, and at this moment, the force exerted on end P (F_{torque}) is shown in the inset. When the substrate cannot provide sufficient friction force, the horizontal component F_{torque}^{P} will actuate the particle chain to move forward. Therefore, a net translation of PQ will be induced

from T3 to T4, as well as the oscillating plane (from A to B at T4). The schematic analysis explains the experimental phenomenon well. In this work, the swarm is actuated by the field with pitch angles, and based on the mechanisms, net translation can be realized.

Supplementary Note 12: Translational velocities with a fixed oscillation ratio: The relationship between the translational velocity and pitch angles with different oscillating frequencies for mode I and mode II are shown (Supplementary Fig. 14). Compared with the case of mode I, the change of the translational velocity with pitch angle is relatively irregular in mode II. As a result, mode I has more advantages in motion control and velocity optimization compared with those of mode II.

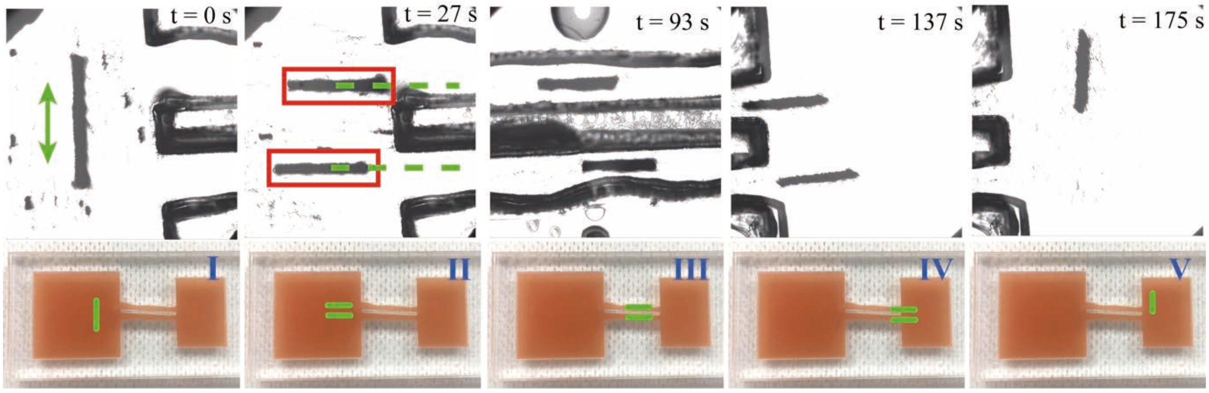

Supplementary Figure 15: The demonstration of an RPNS splitting into two subswarms, passing through two channels simultaneously, and finally remerging into one swarm again. The green ribbons schematically represent the RPNS before and after passing through the channels. The green arrows show the desired path for each subswarm. The oscillation ratio during the locomotion is 3.5 and that used for the pattern elongation is 6. The magnetic field strength is 10 mT, the actuating pitch angle is 8°, and the oscillating frequency is 30 Hz. The scale bar is 800 µm.

Supplementary Note 13: Demonstration of an RPNS moving in another confined environment: In highly confined channels, the width of an RPNS may exceed the width of the channels, and it is possible for the microswarm to be stuck. Therefore, in some cases, one RPNS is required to split into several subswarms first and then, to pass through different channels with navigation. The split subswarms can be merged into one RPNS again afterwards. The experimental validation is demonstrated in Supplementary Fig. 15. Initially, an RPNS is located at the entrance of two channels and is elongated by increasing the oscillation ratio. Then, the swarm is actuated to turn 90◦ to be parallel to the channels with a rotational velocity of 0.35 rad/s, and then splits into two subswarms. Next, both of the

subswarms are navigated to pass through the channels (Supplementary Fig. 15, 27 s - 137 s). Because the patterns of the channels are irregular with varied widths, the navigation process also relies on the high stability of RPNSs, which allows the contact between the swarm and the sidewalls. Finally, when both of the subswarms pass through the channels, they merge into one RPNS (Supplementary Fig. 15, 137 s - 175 s).

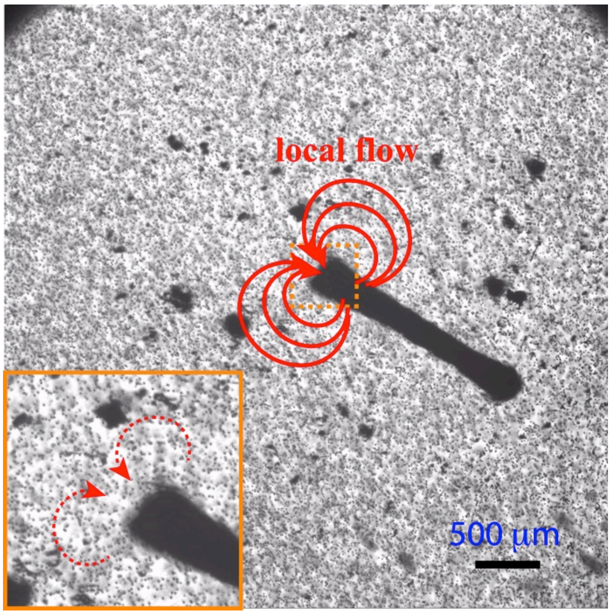

Supplementary Figure 16: The local flow induced by the tips of an RPNS, labeled by tracer particles with an average diameter of 100 μm.

Supplementary Note 14: Pumping effect: We investigate the flow field induced by the tip of an RPNS, by applying tracer particles, as shown in Supplementary Fig. 16. In Supplementary video 5, an RPNS has pumping effects that the tracer particles are attracted into its tips, and then pumped out from the other parts of the microswarm.

Supplementary Note 15: Paramagnetic nanoparticles.

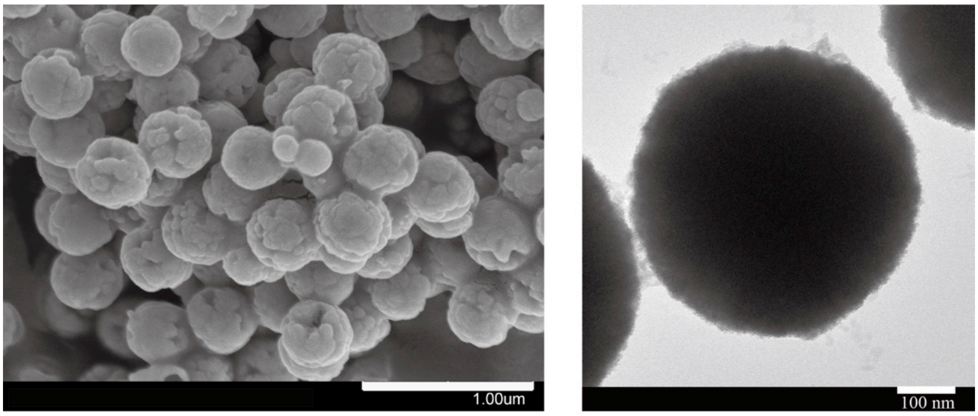

Supplementary Figure 17: a, The SEM image of a cluster of the paramagnetic nanoparticles used in our work. **b**, The TEM image of one particle. The nanoparticles have an average diameter of 500 nm.

Supplementary Note 16: Experimental setup. Supplementary Fig. 18 shows the Helmholtz electromagnetic coil setup [6] in our work.

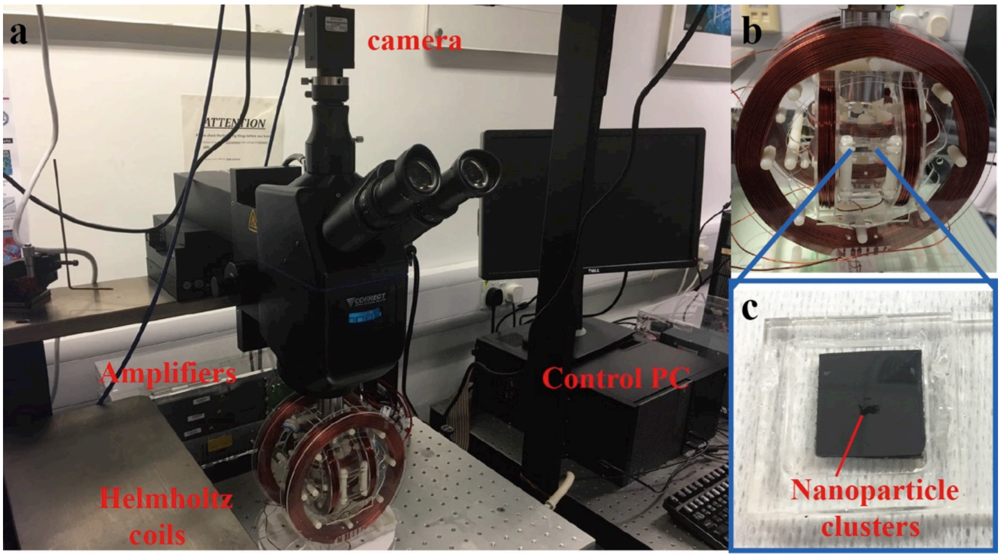

Supplementary Figure 18: a, Experimental setup system. **b**, Detailed view of a set of three-axis Helmholtz electromagnetic coils. **c**, Detailed view of the tank and nanoparticle clusters.

Supplementary References

1. van Reenen A., de Jong, A. M., den Toonder J. M. & Prins M.W., Integrated lab-on-chip biosensing systems based on magnetic particle actuation–a comprehensive review, *Lab Chip*, vol. 14, no. 12, pp. 1966–1986, 2014.

2. Landau L. D., Bell J. S., Kearsley M. J., Pitaevskii L. P., Lifshitz E. M. & Sykes J. B., Electrodynamics of continuous media. *elsevier*, 2013.

3. Singh H., Laibinis P. E. & Hatton T. A., Rigid, superparamagnetic chains of permanently linked beads coated with magnetic nanoparticles. Synthesis and rotational dynamics under applied magnetic fields. *Langmuir*, vol. 21, no.12: 11500-11509, 2005.

4. Schill R. A., General relation for the vector magnetic field of a circular current loop: a closer look, *IEEE Trans. Magn.*, vol. 39, no. 2, pp. 961–967, 2003.

5. Camassa R., Leiterman T. J. & McLaughlin R. M., Trajectory and flow properties for a rod spinning in a viscous fluid. Part 1. An exact solution. *J. Fluid Mech*, 2008, 612: 153-200.

6. Zhou Q., Petit T., Choi H., Nelson B. J. & Zhang L., Dumbbell Fluidic Tweezers for Dynamical Trapping and Selective Transport of Microobjects, *Adv. Funct. Mater.*, Vol. 27(1), 1604571, 2017
