# Supplementary material for: Ultra-extensible ribbon-like magnetic microswarm
Source: Nat Commun. 2018 Aug 21;9:3260. doi: 10.1038/s41467-018-05749-6 (PMC6104072; doi:10.1038/s41467-018-05749-6)
Supplement: Supplementary file 2 — Description of Additional Supplementary Files [file 41467_2018_5749_MOESM2_ESM.pdf]

Description of Additional Supplementary Files

File Name: Supplementary Movie 1
Description: The generation process of a ribbon-like paramagnetic nanoparticle swarm (RPNS). The applied maximum magnetic field strength is 10 mT, the oscillating frequency is 30 Hz, and the amplitude ratio is 3.

File Name: Supplementary Movie 2
Description: The schematic process of the generation of an RPNS. The blue and red parts indicate different magnetizations on nanoparticle chains.

File Name: Supplementary Movie 3
Description: The reversible elongation process of an RPNS with a high aspect ratio. The applied maximum magnetic field strength is 10 mT, and the oscillating frequency is 30 Hz. The amplitude ratio is increased from 3 to 5 for the elongation, and is recovered to 3 for the shrinking.

File Name: Supplementary Movie 4
Description: The merging process of two self-independent RPNSs into one swarm. The applied maximum magnetic field strength is 10 mT, the oscillating frequency is 30 Hz, and the amplitude ratio is 3.

File Name: Supplementary Movie 5
Description: The splitting process of an RPNS into multiple subswarms. The applied pitch angle of the field changes from 0° to 90°, and then, from 90° to 0° again.

File Name: Supplementary Movie 6
Description: The schematic processes of the elongation and shrinkage of an RPNS. The blue and red parts indicate different magnetizations on nanoparticle chains.

File Name: Supplementary Movie 7
Description: The investigation of the swarm behaviour of RPNSs when encountering different boundary conditions, including a circular plane, a planar plane, an angled obstacle, and collision with sidewall.

File Name: Supplementary Movie 8
Description: The locomotion of RPNSs in two channels. The actuating pitch angle is 8°, and the amplitude ratio for the pattern elongation is 6.

File Name: Supplementary Movie 9
Description: The non-contact micromanipulation of four random-distributed polystyrene microbeads into a linear array.

File Name: Supplementary Movie 10
Description: The investigation of the flow field generated at the tips of a RPNS, by applying tracing particles.
